# Impact of Apical Shaping Diameter on Bacterial Load Reduction in Multispecies Infected Root Canals

**DOI:** 10.1155/ijod/8479056

**Published:** 2026-04-20

**Authors:** Abdelkarim El Osta, Marc Krikor Kaloustian, May Mallah, Roula El Hachem, Alfred Naaman, Issam Khalil, Mostafa Yammout, Carla Zogheib

**Affiliations:** ^1^ Department of Endodontics, Saint-Joseph University, Beirut, Lebanon, usj.edu.lb; ^2^ Department of Bacteriology, Saint Joseph University, Beirut, Lebanon, usj.edu.lb; ^3^ Department of Pedodontics, Saint-Joseph University, Beirut, Lebanon, usj.edu.lb

**Keywords:** bacteria biofilms, bacterial infections, diode laser, disinfection, endodontics, *Enterococcus faecalis*, microbial interactions, root canal preparation

## Abstract

**Aim:**

To evaluate the impact of larger apical shaping and the efficacy of different disinfection protocols on increasing the bacterial reduction.

**Methods:**

*Enterococcus faecalis* (E.F.) was cultivated on plate count agar (PCA) and then transferred to test tubes containing brain heart infusion (BHI) broth. Then, *Pseudomonas aeruginosa* (P.A.), *Candida albicans* (C.A.), and *Proteus mirabilis* (P.M.), were introduced one after the other in a specific timeline. After that, 70 lower incisors were infected by this bacterial suspension. Afterwards, these teeth were randomly divided into seven groups for different chemomechanical preparations: group A1 (25/3% + XP endo finisher [XPF]), A2 (25/3% + diode laser), B1 (30/3% + XPF), B2 (30/3% + diode laser), C1 (30/5% + XPF), C2 (30/5% + diode laser), and D (15/3% + syringe irrigation only). Bacterial counting was done on the PCA plates that showed us all the colonies, so it referred to the total number of microorganisms. The Wilcoxon test and the Bonferroni post hoc test were used to obtain a pairing comparison between the various groups.

**Results:**

All groups induced a significantly higher bacterial reduction compared to the control group (*p* < 0.001). There was no significant difference between group A1 and A2 (*p* = 0.45), nor between B1 and B2 (*p* = 0.22), nor between C1 and C2 (*p* = 0.32), nor between B1 and C1 (*p* = 0.58), nor between B2 and C2 (*p* = 0.74). B1 was significantly more effective than A1 (*p* < 0.001) and B2 was significantly more effective than A2 (*p* < 0.001).

**Conclusions:**

To remove as much bacteria as possible, it is preferred to increase the diameter of the apical part. Even with the usage of a diode laser or XPF, these methods perform better when the apical part is enlarged.

## 1. Introduction

Endodontics faces pulpal and periapical diseases, necessitating a comprehensive approach including mechanical debridement, chemical cleaning, and three‐dimensional filling [1, 2]. Bacteria and biofilm in root canal walls can enter dentinal tubules, ramifications, and isthmus between canals; these areas are mostly found in the canal’s apical part [3], which makes it a crucial area for endodontists due to its complexity [4, 5]. Over the past 20 years, the debate over the perfect apical preparation dimension in endodontics has been ongoing [6]. Some argue for better disinfection and better outcomes with larger dimensions, while others believe no significant difference exists between raised and unraised apical part sizes [6]. Various techniques are used to enhance the disinfection of the root canal [7], including the use of the XP endo finisher (XPF) (XPF; FKG Dentaire SA, La Chaux‐de‐Fonds, Switzerland). This tool agitates the irrigant in the canal, removing more bacteria in difficult‐to‐reach areas, enhancing irrigation effectiveness in endodontics [8].

The use of diode lasers in root canal therapy has grown significantly due to their antibacterial properties by heating the fiber tip and increasing the efficiency of sodium hypochlorite [9, 10]. They also reduce bacteria in the deep layers of infected root canal dentin [9]. To this day, the term apical enlargement can be confusing whether, it is the increase of the diameter or the taper of the root canal’s apical part. Additionally, while both XPF and diode laser are used as adjunctive methods to improve root canal disinfection, their differing mechanisms of action warrant direct comparison. However, evidence comparing their antibacterial efficacy and the influence of apical enlargement on their effectiveness remains limited. Therefore, the aim of this study was to evaluate the impact of larger apical shaping and the efficacy of diode laser and XPF on increasing the bacterial reduction.

The first null hypothesis was that there would be no statistically significant difference between the use of XPF and diode laser techniques on intracanal bacterial reduction. On the other hand, the second null hypothesis was that there would be no statistically significant difference observed with increasing the diameter of the shaping instrument’s tip on intracanal bacterial reduction. Last, the third null hypothesis was that there would be no statistically significant difference observed with increasing the shaping instrument’s taper on intracanal bacterial reduction.

## 2. Methodology

**Figure 1:**
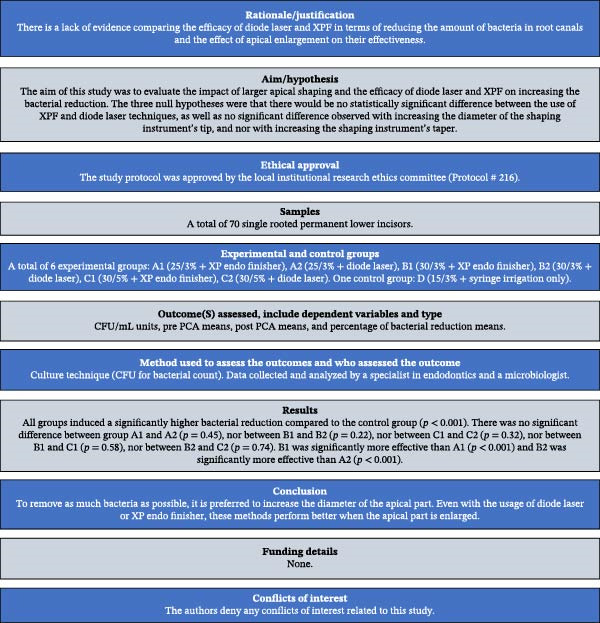
PRILE 2021 flowchart [11].

This in vitro study was reported in accordance with the relevant items of the STROBE guidelines, where applicable, and followed the PRILE 2021 guidelines for reporting laboratory‐based studies in endodontology (Figure 1) [11, 12]. This study was self‐funded. This study protocol was approved by the local institutional research ethics committee (USJ‐2023‐216).

## 3. Sample Selection

A large number of single‐rooted permanent lower incisors, freshly extracted for periodontal reasons, were taken. Cone beam computed tomography (CBCT) (DENTSPLY, Sirona) for these teeth and a visual check on microscope were done to ensure many of the inclusion and exclusion criteria based on several articles [8, 13, 14]. The included teeth followed these criteria: single‐rooted lower incisor, a type 1 canal (per Vertucci’s categorization), not treated endodontically, and teeth with straight canals. Conversely, teeth that met any of the following exclusion criteria were not allowed: root canals with internal or external resorption, calcifications in the root canal, deep caries on the root, roots with cracks or fractures, teeth with immature apex, or >10° root canal curvature [15].

The CBCT taken for these teeth, to assess the initial morphological parameters, had a field of view of 80 mm × 80 mm and a voxel size of 200 mm. The exposure time of 12 s and slice thickness of 0.4 mm with 90 kV and 10 mA were designated [16, 17]. Three cross sections were observed for each tooth: −1, 3, and −5 mm from the apex; each section was aligned with the horizontal plane where the fixed tooth was placed. The images were evaluated using the Sidexis scanner software (DENTSPLY, Sirona). To estimate the volume of the canal, the number of voxels from the outer surfaces of the canal was calculated. Only teeth with slightly similar canal volumes were selected [16, 17].

To determine the sample size, a power analysis for 3 × 2 ANOVA (fixed effects, special, main effects, and interactions) was conducted using 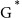
Power software 3.1.9.7 for Windows (Heinrich Heine, Universitat Düsseldorf, Düsseldorf, Germany). A power of 0.8, an alpha level of 0.05, seven groups, and a numerator degree of freedom of two were considered, and a large conventional effect size of 0.4 was assumed.

The minimum sample size required was 10 teeth per subgroup.

## 4. Sample Preparation

In order to reach a length of 16 mm for each root, the coronal portions of the teeth were cut using sterile diamond discs (Prodont Holliger, Vence, France) perpendicular to their longitudinal axes under cooling water. All samples were immersed in an ultrasonic bath (Fisher Scientific Inc., Schwerte, Germany) to get rid of the cutting dust and dirt. Following that, the length of each canal was verified when the #10 K‐Flexofile (Dentsply, Maillefer) became visible at the apical foramen, ensuring the patency of the root canals. The measured length was then deducted by 0.5 mm. Next, the glidepath was finished up to the working length using a #15/3% Minikut rotary file (PlanB dentistry, Germany) mounted on an electric motor (E‐connect S, Eighteeth, Changzhou, China).

After performing the glidepath, the diameter of the canal at 1 mm of the WL should be Ø18, and Ø21 at 2 mm, so the #20 K‐Flexofile should be able to reach between −1.5 and −2 mm of the WL without being able to reach the apex or 1 mm.

Consequently, after doing the glidepath, if the #15 K‐Flexofile directly reached the apex but the #20 K‐Flexofile reached 2 mm or shorter, the tooth was kept. However, if the #20 K‐Flexofile reached the apex or 1 mm, the tooth was excluded from the study.

In order to remove residual pulp tissues and smear layer, root canals were irrigated with 5.25% NaOCl, saline, and 17% EDTA (Vista Dental Products, Racine, WI).

To prevent material extrusion or leakage during root canal preparation and sample collection, the apical 3 mm of each root, including the foramen, was covered with acrylic resin (Dyract AP, DeTrey, Dentsply, UK).

All these steps were done under a microscope at a magnification of ×16.

## 5. Specimen Sterilization

Aliquots of 5 mL sterile brain heart infusion (BHI) solution were added to flasks containing each specimen in the Microbiology Department of Saint Joseph University. Next, agitation was performed in an ultrasonic bath for ~15 min to facilitate the penetration of BHI into the root canal. Following the completion of the agitation, the samples were autoclaved for 30 min at 121°C. After that, the samples were incubated in 10% CO_2_ at 37°C for 48 h.

Visual inspection of the BHI in the flasks was done on a regular basis in order to assess and remove any specimens that were turbid, a sign that bacteria had survived.

## 6. Bacterial Culturing and Specimen Inoculation

The *Enterococcus faecalis* (E.F.) strains (ATCC 29212), obtained from the Microbiology Department of Saint Joseph University, were cultured aerobically on plate count agar (PCA) at 37°C for 48 h. Then, these strains were grown in the liquid nutrient medium BHI broth + 5% saccharose and incubated at 37°C for 24 h in a shaker incubator, followed by 24 h of static incubation in 10% CO_2_. The suspension was calibrated using the CristalspecTM device to achieve a turbidity of 0.5 on the McFarland scale (1.5 × 10^8^ bacterial colonies/mL) (colony‐forming unit [CFU]/mL).

In this experiment, bacterial growth and biofilm formation were planned to be performed outside the canals; therefore, five sterile test tubes were each filled with 10 mL of sterile BHI broth, 5% saccharose, and dental debris.

After that, using a sterile micropipette, 1 mL of the E. F. suspension was added into each of the five tubes, then 1 mL of the *Pseudomonas aeruginosa* (P.A.) suspension was added on the 8th day, followed by 1 mL of *Candida albicans* (C.A.) after 2 days, and finally, 1 mL of the *Proteus mirabilis* (P.M.).

The P.A. (ATCC 27853) strains and the P.M. (ATCC 12453) were cultured aerobically on PCA, but the C.A. (ATCC 10231) strains were grown on Yeast Glucose Chloramphenicol (YGC). The procedures used to prepare the E.F. suspension were identical to the ones used to prepare the P.A., P.M., and C.A. suspensions. All the tubes, containing the multispecies biofilm, were incubated for 2 days at 37°C after the addition of all the microorganisms.

Next, the root canals were dried using sterile paper tips. Consequently, using a sterile micropipette, 10 μL of the bacterial suspension made in the five tubes was injected into each canal, and using a #10 K file, the root canal’s content was mixed with peripheral motions up to working length five times.

The teeth that had been infected were incubated for a week at 37°C with 10% CO_2_, soaked in sterile BHI solution with saccharose, and 10 μL BHI was added every second day of incubation; after each addition of BHI, the teeth were placed on a shaker for 30 min.

## 7. Preoperative Sampling

In order to dissociate the biofilm in the canal, a precurved #10 K file was used to scrape the dentinal walls in 10 movements of back‐and‐forth. The fluids from the root canals were collected using three sterile paper points for 5 min each, and then they were promptly deposited in sterile Eppendorf tubes containing 0.5 mL BHI, then dilutions of 1/10, 1/100, and 1/1000 were made. Following a 15‐min duration, the mixture was vortexed for 30 s, then a sterile micropipette was used to extract 50 μL of the liquid medium from the Eppendorf tubes, and then cultivated for 3 days at 37°C in 10% CO_2_ on PCA, YGC, and Uriselect. After incubation, when visible colonies began to emerge on the plate, the number of CFUs in the initial sample was counted and calculated. For pre‐ and postoperative sampling, YGC Petri dishes showed the colonies of C.A. (yellow spots); Uriselect Petri dishes showed the colonies of E.F. (green spots) and P.M. (white spots); and PCA Petri dishes showed, nonspecifically, the three bacteria and the yeast. That is why the total number of bacteria was taken from PCA Petri dishes.

## 8. Specimen Grouping and Chemomechanical Preparation

The roots were randomly divided into three experimental groups (A, B, and C, *n* = 20 each) and one control group (D, *n* = 10). After that, in the three experimental groups, the root canals being patent, as mentioned before, instrumentation was performed up to the working length using Minikut rotary instruments (PlanB Dental, Germany) mounted on the electric motor (E‐connect S, Eighteeth, Changzhou, China) with programmed torque control and speed settings. The #25/3% rotary file was used to shape the root canals in group A, the #30/3% rotary file was used in group B, and the #30/5% rotary file was used in group C. During the procedure, the shaping instrument achieved the working length in three phases at least; the first entry was done until 8 mm with light lateral pressure applied to the canal walls, then the second entry was done until 12 mm, and the third entry achieved the working length (16 mm). Between files, patency at working length was confirmed by using a #10 K‐file, and canals were irrigated with 5.25% NaOCl with a 30‐gauge side‐vented (blind‐ended) endodontic irrigation needle (Kerr‐Hawe, Switzerland). The volume of irrigant flushed after each file is 3 mL, adopting the same distance between the needle and the foramen: 4 mm.

Consequently, each group was divided into two subgroups:

A1, B1, and C1, each including 10 root canals, were subjected to this disinfection protocol: 5 mL of 5.25% NaOCl, activated with XPF (FKG); then 5 mL of saline; then 5 mL of 17% EDTA, activated with XPF; then 5 mL of saline; then 5 mL of 5.25% NaOCl, also activated; and finally a final rinse of distilled water. The XPF operated 2 mm far from the WL with a speed of 1000 rpm.

A2, B2, and C2, each including 10 root canals, were subjected to this disinfection protocol: 5 mL of 5.25% NaOCl, followed by a laser irradiation; then 5 mL of saline; then 5 mL of 17% EDTA, followed by a laser irradiation; then 5 mL of saline; then 5 mL of 5.25% NaOCl, followed by a laser irradiation; and finally a final rinse of distilled water.

The laser irradiation was done with 2.5 W power, used in gated mode (10 ms ON–10 ms OFF) for 5 s with a resting time of 10 s repeated three times, so the total irradiation time was 15 s for each solution, while inserting the fiber optic tip 2 mm far from the WL.

In the control group, roots were inoculated but received neither mechanical preparation nor irrigation procedures.

It is important to note that all the procedures were carried out by the same operator (A.O.) to reduce variables; only the pre‐ and postoperative CFU counting was done by two specialized operators (A.O. and M.Y.) in order to finish all the counting on the same day.

## 9. Postoperative Samplings

The procedures used to do the preoperative samplings were identical to the ones used to do the postoperative samplings.

**Figure 2:**
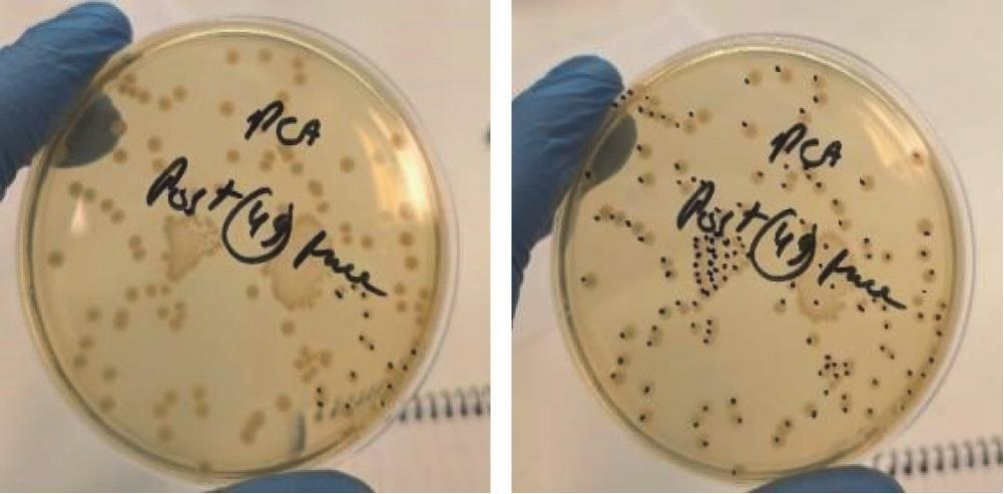
PCA plates showing the CFU counting.

After incubation, when visible colonies began to emerge on the plate, the number of CFUs in the initial was calculated (Figure 2).

## 10. Results

**Figure 3:**
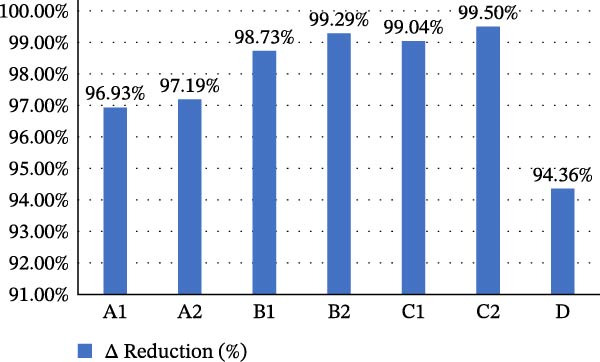
Percentage of bacterial reduction means for each group.

A total of 70 teeth were included and equally distributed among seven experimental groups. Results of the mean pre‐ and postoperative PCA for each group and the percentage of bacterial reduction means (± SD) are presented in Table 1. Overall, all experimental groups demonstrated a significantly greater bacterial reduction compared with the control group (15/3% preparation with syringe irrigation only) (*p* < 0.001) (Table 2). Although bacterial reduction reached values as high as 99.5%, complete eradication of microorganisms was not achieved by any of the tested protocols (Figure 3).

**Table 1:** Mean pre‐ and postoperative PCA for each group and the percentage of bacterial reduction means.

Group	Mean pre‐PCA 1/1000	Mean post‐PCA 1/1000	Mean percentage of bacterial reduction
A1	4.40 × 10^6^ (±3.03 × 10^6^)	1.35 × 10^5^ (±7.64 × 10^4^)	96.93% (±0.69)
A2	3.90 × 10^6^ (±2.47 × 10^6^)	1.10 × 10^5^ (±7.31 × 10^4^)	97.19% (±1.01)
B1	2.88 × 10^6^ (±2.20 × 10^6^)	3.66 × 10^4^ (±3.15 × 10^4^)	98.73% (±0.25)
B2	2.70 × 10^6^ (±2.16 × 10^6^)	1.91 × 10^4^ (±1.74 × 10^4^)	99.29% (±0.18)
C1	3.30 × 10^6^ (±1.89 × 10^6^)	3.16 × 10^4^ (±1.84 × 10^4^)	99.04% (±0.30)
C2	4.50 × 10^6^ (±3.21 × 10^6^)	2.25 × 10^4^ (±1.40 × 10^4^)	99.50% (±0.12)
D	3.6 × 10^6^ (±1.5 × 10^6^)	2.03 × 10^5^ (±9.8 × 10^4^)	94.36% (±1.4)

**Table 2:** Statistical comparison between the percentage of bacterial reduction means of groups A1, A2, B1, B2, C1, C2 and group D.

Parameters	Groups						
	A1	A2	B1	B2	C1	C2	D
Δ Reduction (%) (± SD)	96.93% (±0.69)	97.19% (±1.01)	98.73% (±0.25)	99.29% (±0.18)	99.04% (±0.30)	99.50% (±0.12)	94.36% (±1.4)
*p*‐Value	<0.001 ^∗^	<0.001 ^∗^	<0.001 ^∗^	<0.001 ^∗^	<0.001 ^∗^	<0.001 ^∗^	<0.001 ^∗^

^∗^To enhance the importance of the results.

When comparing the two adjunctive disinfection methods, no statistically significant difference was observed between XPF and diode laser within the same apical preparation parameters. Specifically, no significant differences were found between groups A1 (25/3% + XPF) and A2 (25/3% + diode laser) (*p* = 0.45), B1 (30/3% + XPF) and B2 (30/3% + diode laser) (*p* = 0.22), or C1 (30/5% + XPF) and C2 (30/5% + diode laser) (*p* = 0.32) (Table 3).

**Table 3:** Statistical comparison between the percentage of bacterial reduction means of groups A1 and A2, B1 and B2, C1 and C2 (according to the disinfection protocol).

Parameters	Groups					
	A1	A2	B1	B2	C1	C2
Δ Reduction (%) (±SD)	96.93% (±0.69)	97.19% (±1.01)	98.73% (±0.25)	99.29% (±0.18)	99.04% (±0.30)	99.50% (±0.12)
*p*‐Value	0.45		0.22		0.32	

Increasing the apical diameter from #25 to #30 resulted in a statistically significant improvement in bacterial reduction, regardless of the adjunctive disinfection method used. Groups B1 (30/3% + XPF) and B2 (30/3% + diode laser) showed significantly greater bacterial reduction compared with their respective #25 counterparts, A1 and A2 (*p* < 0.001) (Table 4).

**Table 4:** Statistical comparison between the percentage of bacterial reduction means of groups A1 and B1, A2 and B2 (according to the shaping instrument’s tip diameter).

Parameters	Groups			
	A1	B1	A2	B2
Δ Reduction (%) (±SD)	96.93% (±0.69)	98.73% (±0.25)	97.19% (±1.01)	99.29% (±0.18)
*p*‐Value	<0.001 ^∗^		<0.001 ^∗^	

^∗^To enhance the results.

In contrast, increasing the taper from 3% to 5% at a constant apical diameter of #30 did not result in a statistically significant difference in bacterial reduction. No significant differences were observed between groups B1 (30/3% + XPF) and C1 (30/5% + XPF) (*p* = 0.58), nor between groups B2 (30/3% + diode laser) and C2 (30/5% + diode laser) (*p* = 0.74) (Table 5).

**Table 5:** Statistical comparison between the percentage of bacterial reduction mean of groups B1 and C1, B2 and C2 (according to the shaping instrument’s taper.

Parameters	Groups			
	B1	C1	B2	C2
Δ Reduction (%) (±SD)	98.73% (±0.25)	99.04% (±0.30)	99.29% (±0.18)	99.50% (±0.12)
*p*‐Value	0.58		0.74	

CFU counts obtained from PCA represented the total viable microbial load. Mean percentages of bacterial reduction for each group were calculated based on pre‐ and post‐instrumentation CFU values:

Mean pre-PCA−mean post-PCAMean pre-PCA×100.

## 11. Discussion

In clinical practice, periapical lesions are often caused by bacteria, which form multicellular biofilms in the canal system [18]. Despite intracanal disinfection and a changed environment, bacteria can still be detected in samples retained after root canal treatment, highlighting the need for effective treatment [19].

This study aimed to evaluate the impact of larger apical shaping and the efficacy of diode laser and XPF on increasing the bacterial reduction.

E.F., a biofilm‐forming organism, is responsible for endodontic treatment failures [14], prompting extensive research on treatment efficacy [20]. This study focuses on canal infections caused by a synergy of microorganisms, including E.F., P.A., P.M., and C.A., which are often found in necrotic pulpal infections [21]. C.A., the most frequently cited fungus in root canal infections [22], attaches to dental dentin, forms biofilms, and infiltrates dentinal tubules, making them difficult to eradicate, increasing root canal infections [23]. This experiment selected P.M. and P.A. due to their strong synergy and biofilm formation [24].

The study suggests that the introduction of bacteria in a specific order may have fostered growth and synergistic interactions between P.A., E.F., and subsequent species [25, 26]. This could potentially facilitate future bacterial attachment and growth, influencing the biofilm structure and increasing the overall bacterial count. The study suggests that complex relationships, metabolic exchanges, and adhesion mechanisms may contribute to the increase in bacteria [19], such as E.F. and P.A. coaggregating, potentially leading to endodontic infections and resulting in a higher microbial population [19, 26].

The study used the microbiological culturing method to evaluate root canals. However, this method has limitations, as it does not release adhering microorganisms. To dislodge the biofilm and remove any remaining microorganisms, physical separation processes like scraping with a K‐file #10 are used [26]. Although this treatment is likely beneficial for teeth with simple canal architecture, it is unclear what proportion of the microbial load is removed from the canal due to its lack of validation [26]. In addition, microbiological culturing is a labor‐intensive method used in laboratories to accurately count CFUs, but it is limited to culturable microorganisms and requires sterile media preparation [26, 27].

This study conducted biofilm formation outside the teeth, following the same methodology for contaminating canals [26, 28–31]. After adding microorganisms in the same order in five test tubes containing BHI, a bacterial solution with a visible multispecies biofilm was formed in each tube. In this study, a staining technique described by Mohmmed et al. [32] was used to confirm the biofilm formation. The tubes were cleaned three times with phosphate buffered saline (PBS) to get rid of any loose cells, then methanol was added, and after 30 min, staining with 1% crystal violet (CV) was done for 30 min. After rinsing with distilled water, the biofilm was confirmed (Supporting Information Figure 1). Before staining, 10 µl of the solution was diluted and cultured on YGC, Uriselect, and PCA to confirm the presence of all four microorganisms. The study used CBCT imaging and K‐files to select teeth with similar canal volume, but µCT imaging was found to be the most accurate method for confirming exact canal volume [26].

In this study, all groups did a significantly better job than the control group (Table 2). Despite that syringe irrigation after the glidepath with the instrument 15/3% showed the lowest percentage of bacterial reduction (94.36%) (group D), this conventional method is still widely used by dentists [33]. Group D’s lowest bacterial reduction percentage is due to the canal’s lack of mechanical preparation, hindering the needle tip’s reach for effective disinfection [34]. Correspondingly, several ex vivo studies show syringe irrigation effectively removes bacteria, biofilm, hard tissue debris, and soft tissue remnants from teeth with a single root canal and simple anatomy [35–38]. On the other hand, research that came to the opposite conclusion typically did not widen the canals to the proper size or position the needles too far from the WL [39–42]. Therefore, irrigation activation techniques might be useful in situations where the anatomy is more complex due to the limited flow generated by anatomical abnormalities such as fins, anastomoses, and lateral canals [38, 42, 43].

When comparing the two disinfection methods (Table 2), diode laser and XPF showed no statistically significant difference in their ability to reduce intracanal bacterial load, leading to the acceptance of the first null hypothesis. This lack of difference may be attributed to several factors. First, the sample size may not have been sufficient to detect small differences between two adjunctive techniques that both act as enhancements to conventional chemomechanical preparation. Second, although their mechanisms differ, both methods may exhibit comparable limitations in biofilm penetration depth within dentinal tubules, particularly in the apical third. In addition, the activation protocols used—including laser parameters, optical fiber diameter, and XPF activation time in the presence of sodium hypochlorite—may have resulted in similar levels of irrigant penetration and antimicrobial effect. Finally, apical enlargement itself may play a dominant role in bacterial reduction, potentially masking subtle differences between the adjunctive disinfection methods.

These outcomes were linked by researchers to XPF’s mechanical action and irrigation activation [44]. Another study found that XPF with NaOCl performed well in removing bacterial biofilms by getting to the difficult‐to‐reach areas [8]. Nevertheless, the use of XPF must work along with NaOCl in order to eradicate E.F. from the root canals [45].

The diode laser has been shown to improve sodium hypochlorite penetration, leading to improved root canal system disinfection [46]. Laser irradiation following chemomechanical irrigation outperformed NaOCl irrigation alone in terms of root canal disinfection and E.F. eradication [47]. Using a diode laser to occlude dentinal tubules, particularly in the apical third area, following smear layer ablation, decreases the risk of reinfection [48]. The diode laser was used with a 200 µm optical fiber to allow adequate energy delivery within the root canal system, which is suitable for endodontic disinfection. In addition to facilitating access to the apical third, diode laser irradiation exerts a photothermal antibacterial effect, enhances sodium hypochlorite penetration into dentinal tubules, and contributes to smear layer modification, thereby improving overall root canal disinfection [49].

With the same 3% taper (Table 2), increasing the diameter of the shaping instrument’s tip from #25 to #30 induced significantly better bacterial reduction, irrespective of the disinfection method used; this means that the second null hypothesis has been rejected. This is in concordance with the results of articles that mentioned how the apical enlargement can lead to a better microbial reduction [50–52]. Even with the usage of different activation methods, apical enlargement can increase the effectiveness of these techniques [51, 53, 54].

With the same #30 tip (Table 2), increasing the taper of the shaping instrument from 3% to 5% did not induce significantly better bacterial reduction, irrespective of the disinfection method used; this means that the third null hypothesis has been accepted. This study found that a taper increase is not always necessary when using a disinfection method alongside passive irrigation. However, both XPF and diode laser were more efficient when the apical diameter was increased to #30. The XPF, with a #25 diameter, benefits from a wider working area when the root canal is enlarged to #30, improving its mechanical cleaning effect [8, 51]. The diode laser, when its small‐diameter optical fiber reaches the apical zone, can enhance the antimicrobial efficiency of sodium hypochlorite by allowing deeper energy delivery and improved interaction with the irrigant [55]. Increasing the apical diameter facilitates irrigant exchange, improves flow dynamics, and enables deeper penetration of both sodium hypochlorite and laser energy into the apical third and dentinal tubules. In contrast, increasing taper without sufficient apical enlargement may have a limited effect on apical irrigant replacement and laser accessibility, which may explain why most studies emphasize apical diameter rather than taper when aiming to improve bacterial reduction [56, 57].

In this study, the efficiency and uniformity of the detachment process inside the canals are called into question in the lack of visible confirmation using methods like confocal or scanning electron microscopy (SEM). Imaging methods could be used in future research to provide a more accurate measure of biofilm dissociation. The capacity of imaging to focus on a particular root canal location and assess the effectiveness of a material on that particular area makes it beneficial as well. For example, in the study of Azim et al. [39], the authors used the confocal laser scanning microscope (CLSM) in order to check the bacterial reduction at the coronal, middle, and apical segments at a 50‐μm depth of the dentinal tubules. Likewise, in the study of Aoun et al. [58], the authors used the SEM to evaluate their results at the coronal, middle, and apical levels of the root canal.

In addition, future articles could discuss the effectiveness of the diode laser and XPF in reducing the bacterial amount in curved and oval canals.

Another limitation of this study is the usage of paper points to gather root canal samples, which may underestimate the true bacterial load and may not be effective in detecting microbiological loads in anatomical irregularities [59, 60]. Cryopulverization has been suggested as a solution to these drawbacks because it offers a more complete sample from the whole root canal system [21].

Since this study is an in vitro study, its clinical significance can be doubtful to some readers since in our daily practice, a lot of factors can change. Therefore, the present findings should be interpreted as mechanistic evidence supporting adjunctive disinfection strategies rather than as definitive predictors of clinical success.

## 12. Concluding Remarks

This study focused on how the idea of apical enlargement can be tightened to just increase the diameter of the shaping instrument used, without the need for increasing the taper and removing more dentin. Also, this study proves that even when using different disinfection methods such as XPF and diode laser, these methods will be more efficient when enlarging the root canal’s apical part; this information can be a relief to endodontists heading toward a minimally invasive strategy. Although this study provides valuable insights, its limitations underscore the need for caution when applying its findings to clinical practice. Conducting well‐designed randomized controlled trials is necessary to assess the antibacterial effects of diode laser, XPF, and larger apical shaping in vivo. Thus, outcomes that are reported by both clinicians and patients—such as radiographic results, clinical signs and symptom remission, tooth survival, and postoperative pain—should be assessed.

## Author Contributions

**Abdelkarim El Osta**: conceptualization, methodology, validation, investigation, resources, data curation, writing – original draft, visualization. **Carla Zogheib**: conceptualization, methodology, validation, investigation, resources, data curation, writing – review and edition, visualization, supervision, project administration. **Roula El Hachem**: writing – review and edition. **May Mallah**: methodology, data curation. **Issam Khalil**: validation, supervision, project administration. **Alfred Naaman**: validation, supervision, project administration. **Marc Krikor Kaloustian**: writing – review and edition, project administration. **Mostafa Yammout**: investigation.

## Funding

No funding was received for this manuscript.

## Conflicts of Interest

The authors declare no conflicts of interest.

## Supporting Information

Additional supporting information can be found online in the Supporting Information section.

## Supporting information


**Supporting Information** Given the in vitro and laboratory‐based nature of the present study, items specific to clinical observational research were not applicable. In addition, the study reporting adheres to the PRILE 2021 guidelines (Figure 1), which are specifically designed for laboratory studies in endodontology. A STROBE checklist adapted to the present study has been completed accordingly and it is mentioned in the attached document “Extra document about the STROBE Checklist.pdf.”

## Data Availability

The data that support the findings of this study are available upon request from the corresponding author. The data are not publicly available due to privacy or ethical restrictions.
