# Supplementary material for: Impact of Apical Shaping Diameter on Bacterial Load Reduction in Multispecies Infected Root Canals
Source: Int J Dent. 2026 Apr 20;2026:8479056. doi: 10.1155/ijod/8479056 (PMC13094935; doi:10.1155/ijod/8479056)
Supplement: Supplementary file 1 — Supporting Information Given the in vitro and laboratory‐based nature of the present study, items specific to clinical observational research were not applicable. In addition, the study reporting adheres to the PRILE 2021 guidelines (Figure 1), which are specifically designed for laboratory studies in endodontology. A STROBE checklist adapted to the present study has been completed accordingly and it is mentioned in the attached document “Extra document about the STROBE Checklist.pdf.” [file IJOD-2026-8479056-s001.docx]

**Extra document about the STROBE Checklist**

We thank the reviewer for this valuable suggestion. The manuscript has been revised to ensure compliance with the relevant items of the STROBE reporting guidelines, where applicable. Given the in vitro and laboratory-based nature of the present study, items specific to clinical observational research were not applicable. In addition, the study reporting adheres to the PRILE 2021 guidelines, which are specifically designed for laboratory studies in endodontology. A STROBE checklist adapted to the present study has been completed accordingly.

The STROBE checklist is adapted below. Items not applicable to laboratory-based studies are explicitly marked.

**Title and Abstract**

- **Item 1 (a)**: Indicate study design in the title or abstract → **Reported** (in vitro experimental study stated)
- **Item 1 (b)**: Provide informative abstract → **Reported**

**Introduction**

- **Item 2**: Scientific background and rationale → **Reported**
- **Item 3**: Objectives and hypotheses → **Reported**

**Methods**

- **Item 4**: Study design → **Reported** (in vitro experimental design described)
- **Item 5**: Setting → **Reported** (laboratory conditions specified)
- **Item 6**: Participants → **Not applicable** (no human participants)
- **Item 7**: Variables → **Reported** (bacterial reduction, apical size, taper, disinfection methods)
- **Item 8**: Data sources and measurement → **Reported** (microbiological culturing, CFU counts)
- **Item 9**: Bias → **Reported where applicable** (standardization of samples, canal selection)
- **Item 10**: Study size → **Reported** (sample size per group specified)
- **Item 11**: Quantitative variables → **Reported**
- **Item 12**: Statistical methods → **Reported**

**Results**

- **Item 13**: Participants → **Not applicable**
- **Item 14**: Descriptive data → **Reported**
- **Item 15**: Outcome data → **Reported**
- **Item 16**: Main results → **Reported**
- **Item 17**: Other analyses → **Reported where applicable**

**Discussion**

- **Item 18**: Key results → **Reported**
- **Item 19**: Limitations → **Reported**
- **Item 20**: Interpretation → **Reported**
- **Item 21**: Generalizability → **Limited; addressed as in vitro limitation**

**Other Information**

- **Item 22**: Funding → **Reported (**Self-funded**)**

**Reference:**

von Elm E, Altman DG, Egger M, Pocock SJ, Gøtzsche PC, Vandenbroucke JP. The Strengthening the Reporting of Observational Studies in Epidemiology (STROBE) statement: guidelines for reporting observational studies. *Lancet*. 2007;370(9596):1453–1457.

STROBE Initiative. Strengthening the Reporting of Observational Studies in Epidemiology (STROBE) Statement. Available at: https://www.strobe-statement.org
